# Finding the Silver Bullet for Persistent Foramen Hushke

**DOI:** 10.7759/cureus.52791

**Published:** 2024-01-23

**Authors:** Theresa Teoh, Asma Abdullah, Guhan Kumarasamy

**Affiliations:** 1 Otorhinolaryngology, University Kebangsaan Malaysia, Kuala Lumpur, MYS; 2 Otolaryngology, Universiti Kebangsaan Malaysia Medical Centre, Kuala Lumpur, MYS; 3 Otorhinolaryngology and Head and Neck Surgery, Hospital Raja Permaisuri Bainun, Perak, MYS

**Keywords:** ear canal mass, external auditory canal dehiscence, temporomandibular joint prolapse, persistent foramen hushke, tmj herniation

## Abstract

We report a case of persistent foramen Hushke and embark on a literature search from 1990 to 2021. The search was done using electronic databases of PubMed and Google Scholar using the MESH words ‘TMJ herniation’, ‘persistent foramen Hushke’, ‘TMJ prolapse’, ‘EAC dehiscence’, and ‘ear canal mass’. A total of 37 other case reports were included after excluding duplicates and non-English publications. The most common presentations, treatment modalities, complications, and outcomes were discussed. Common presentations include otalgia, tinnitus, otorrhea, and aural fullness. Surgical intervention shows a good outcome, whereas conservative treatment shows a mixed response. Despite the overwhelming success of surgical interventions in treating symptomatic persistent foramen of Hushke, it is still early to establish a guideline to manage these patients, as this condition is rare and presents with variable symptoms. More high-quality studies and a long-term follow-up of the patients may be essential to observe and compare the outcome and recurrence rate of temporomandibular joint (TMJ) herniation.

## Introduction

Persistent foramen Hushke is a relatively common condition, affecting 4.6% to 20% of the population, but only a small number of patients are symptomatic. Due to their rarity and innocuous nature, symptomatic patients rarely present themselves to the hospital [1].

Therefore, there is little literature available besides case reports. Here, we would like to present a case report of a middle-aged lady with persistent foramen Hushke with temporomandibular joint herniation into the external ear canal, basic anatomy and development of the ear canal, and a literature review to compare presentations, management, outcomes, and complications. Independent searches by the authors in the databases of PubMed and Google Scholar were done using the keywords ‘TMJ herniation’, ‘persistent foramen Hushke’, ‘TMJ prolapse’, ‘external auditory canal dehiscence', and ‘ear canal mass’. After excluding non-relevant, duplicate, and non-English studies, 31 papers have been included. Due to the non-homogenous nature of the cases, we proceeded to do a critical review, and the results were interpreted according to our clinical judgment.

## Case presentation

**Figure 1:**
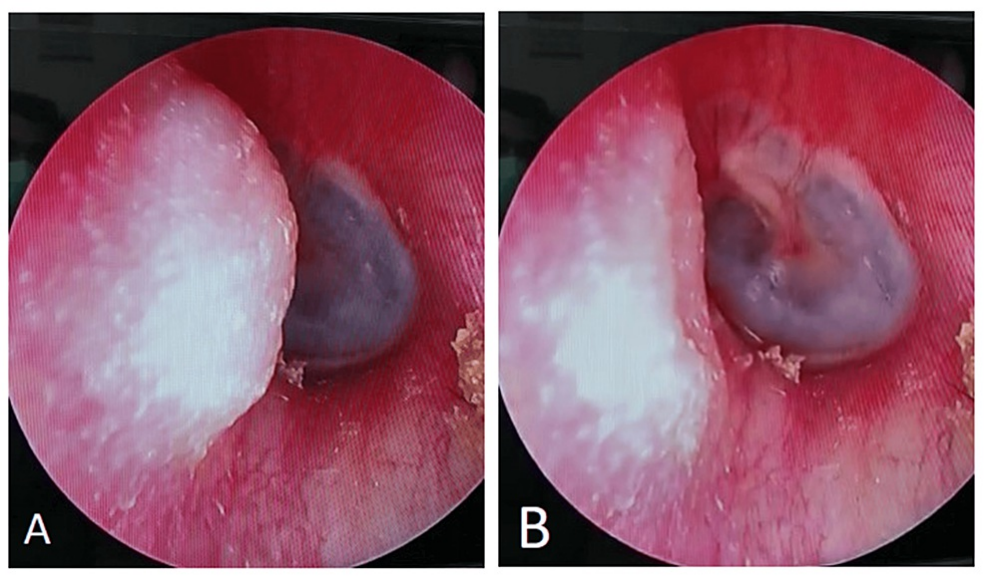
Clinical image showing the TMJ prolapse into external ear canal during opening (A) and closing of mouth (B). Image credit: Theresa Teoh.

A 47-year-old lady with no comorbidities presented to the ENT Department in a district hospital with a complaint of left ear fullness for two months. She mentioned a history of recurrent otitis externa over the left ear for many years, which was treated with over-the-counter medications. Otherwise, she reported no history of tinnitus, hearing impairment, otorrhea, trauma, or aural surgery. Oto-endoscopic examination revealed a small bulging mass over the left anterior wall of the bony external auditory canal. It was approximately 5 mm × 3 mm in dimensions. The mass is soft and lined with normal, healthy skin. The mass protruded when she opened her mouth and retracted when the mouth was closed, as shown in Figure 1.

**Figure 2:**
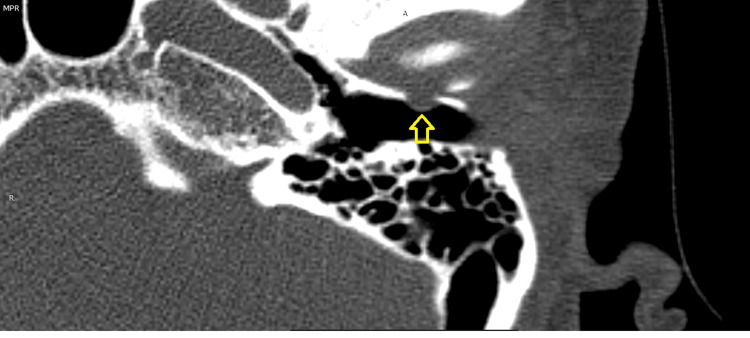
Radiological image of the temporal bone showing defect at anterior wall of bony ear canal. Image credit: Theresa Teoh.

She had no hearing impairment, and the fistula test was negative. With a clinical suspicion of temporomandibular joint (TMJ) involvement, a high-resolution computed tomography (HRCT) scan of the temporal bone was done using MODEL somatom Sensation 16, Siemens AG Forcheim, Germany. A CT scan revealed a small bony defect at the anterior bony canal of the left external auditory canal with protrusion of the TMJ into the ear canal, as shown in Figure 2. No additional abnormalities were identified.

She was counselled for the repair of an external ear canal defect with a titanium plate via a preauricular approach, but she opted for conservative management. She was counselled not to dig her ear, fearing injury to the TMJ capsule leading to otorrhea and infection. The case was followed up every six months up to two years; there was no worsening of symptoms, and it did not disturb her daily activities; hence, no surgical intervention has been done up to this date.

## Discussion

The studies included in our study are summarized in Table 1 [2-32].

**Table 1:** Summaries of studies of Foramen of Hushke reported from year 1990-2021.

	Authors/year	Age/gender	Presentation	Management	Complication	Outcome
1	Ajduk et al. [2]	53/female	Otorrhea ear infection	Open surgery	Nil	Symptoms resolved
2	Akcam et al. [3]	48/female	Otalgia, tinnitus	Open surgery	Nil	Symptoms resolved
3	Anand et al. [4]	51/female	Hearing loss, otalgia, tinnitus	Open surgery	Minor reduction of mouth opening (42 mm)	Symptoms resolved
		46/female	Otalgia, tinnitus	Open surgery	Nil	Symptoms resolved
4	Burlak et al. [5]	52/male	Otorrhea	Open surgery	Not mentioned	Not mentioned
5	Choi et al. [6]	75/female	Tinnitus	Conservative	Nil	Partial improvement
6	Ertugrul [7]	64/female	Tinnitus, otalgia	Conservative	Nil	Partial improvement
7	Fusconi et al. [8]	60/female	Otorrhea infection	Not mentioned	Not mentioned	Not mentioned
8	Heffez et al. [9]	67/female	Otalgia	Conservative	Nil	Partial improvement
		53/female	Otalgia	Conservative	Nil	Not mentioned
9	Kinar et al. [10]	52/male	Otalgia	Conservative	Nil	Not mentioned
10	Kim et al. [11]	53/male	Tinnitus	Conservative	Nil	No improvement
11	Lee and Park [12]	84/male	Otalgia hearing loss otorrhea	Conservative	Nil	Not mentioned
12	Lee and Park [13]	70/male	Tinnitus	Conservative	Nil	No improvement
13	Li and Dai [14]	57/female	Aural fullness, otalgia	Conservative	Nil	Partial improvement
14	Lim et al. [15]	63/female	Tinnitus aural fullness	Conservative	Nil	Not mentioned
		55/male	Otorrhea, tinnitus	Open surgery	Nil	Symptoms resolved
15	Mittal et al. [16]	70/male	Otalgia aural fullness	Conservative	Nil	Not mentioned
16	Moriyama et al. [17]	41/male	Tinnitus	Open surgery	Nil	Symptoms resolved
17	Nakasato et al. [18]	61/male	Tinnitus	Open surgery	Nil	Symptoms resolved
18	Olarinoye-Akorede et al. [19]	28/male	Otalgia hearing loss	Conservative	Nil	Symptoms resolved
19	Prowse et al. [20]	87/female	Otorrhea otalgia	Conservative	Nil	Not mentioned
20	Rushton et al. [21]	59/male	Otorrhea	Open surgery	Nil	Symptoms resolved
21	Ryu et al. [22]	46/male	Tinnitus	Open surgery	Nil	Symptoms resolved
22	Shapiro and Osborn [23]	54/male	Otalgia, tinnitus	Open surgery	Nil	Symptoms resolved
23	Sharma and Dawkins [24]	58/female	Otorrhea	Open surgery	Nil	Symptoms resolved
24	Shin et al. [25]	82/male	Tinnitus	Conservative	Nil	Patient defaulted follow up
		23/female	Tinnitus aural fullness	Conservative	Nil	Symptoms resolved
25	Silva and Collins [26]	5/female	Neck swelling	Open surgery	Nil	Symptoms resolved
		6/female	Neck swelling otalgia otorrhea	Open surgery	Nil	Symptoms resolved
26	Singh et al. [27]	35/male	Otorrhea aural fullness	Open surgery	Suture line wound infection	Symptoms resolved
27	Bernstein et al. [28]	58/female	Aural fullness hearing loss otorrhea	Open surgery	Altered sensation during mastication	Symptoms resolved
28	Weissman et al. [29]	51/female	Otorrhea	Conservative	Nil	No improvement
29	Wong and Al-Hadeethi [30]	43/male	Otalgia aural fullness hearing loss	Conservative	Nil	Partial improvement
30	Xie et al. [31]	69/male	Aural fullness otorrhea otalgia tinnitus	Endoscopic repair	Nil	Symptoms resolved
31	Yoo et al. [32]	66/male	Aural fullness otalgia	Open surgery	Nil	Symptoms resolved
		47/male	Aural fullness otalgia	Open surgery	Nil	Symptoms resolved

The persistent foramen of Huschke is a developmental defect of the antero-inferior bony external auditory meatus. As early as the fifth week of embryological development, an ectodermal diverticulum develops towards the pharynx in between the first and second pharyngeal arches. This forms the future external ear canal. The anterior, inferior, and lower parts of the posterior wall of the external ear canal are formed by the tympanic plate, which lies in between the squamous and mastoid portions of the temporal bone. At the antero-inferior part of this primordial ear canal lies the foramen of Hushke, or tympanicum, which gradually fuses by the age of 5. In some cases, the failure of membranous ossification of the tympanic portion of the temporal bone may give rise to the persistence of the foramen. In a systematic review assessing 2671 patients by cadaveric dissections and radiological assessment, it was found that the foramen of Hushke was present in 21.2% and 8.8% of patients, respectively [1]. The high prevalence of persistent foramen of Hushke suggests a large percentage of patients remain asymptomatic. However, due to the intimate relationship of this canal to the TMJ anterolaterally, the structures of the TMJ can impetuously herniate into the external auditory canal (EAC) through the defect and may produce both clinical and otological symptoms, especially in adults [6,16].

Patients with persistent foramen Hushke may have a combination of aural symptoms. According to our literature review, patients mostly presented with otalgia, followed by tinnitus, otorrhea, and aural fullness. Hearing loss and neck swelling are rare presentations.

**Figure 3:**
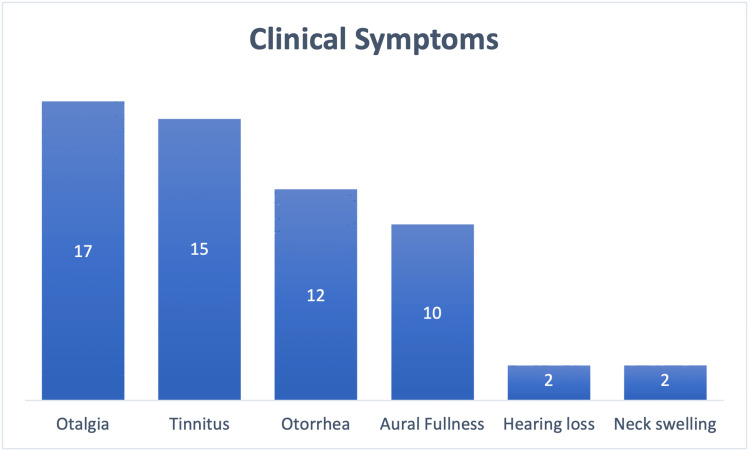
Bar chart showing number of cases and their presenting complaint. Image credits: Theresa Teoh.

In our review of 31 case reports (n = 37 patients), the patients presented with a myriad of presentations, either with a single or a combination of complaints. The most common presentations were otalgia at 45.9% (17/37), tinnitus at 40.5% (15/37), otorrhea at 32.4% (12/37), aural fullness at 27.0% (10/37), hearing loss at 5.4% (2/37), recurrent ear infections at 5.4% (2/37), and neck swelling at 5.4% (2/37) as depicted in Figure 3.

A diagnosis of symptomatic persistent foramen Hushke hinges on a clinical examination and radiographic confirmation of dehiscence. Clinically, a bulging mass at the anterior canal wall may be seen expanding and regressing on jaw movement or the presence of clear fluid in otoendoscopy. Imaging modalities such as conventional or cone beam CT and MRI still remain the gold standard for confirming the diagnosis. HRCT temporal bone imaging has the advantage of identifying small bony defects along the anterior bony EAC wall, which separates the ear canal and the temporomandibular joint. On the other hand, MRI may be better to delineate the contents of soft tissue to differentiate the content of herniation between joint synovium and salivary tissue [33].

Management of this condition depends on the severity of symptoms, the size of the dehiscence, and the patient’s preference. Surgical intervention aims to reduce TMJ herniation and close the dehiscence between the EAC and the TMJ. Both open surgery and, recently, endoscopic approaches have been reported. Open approaches include pre-auricular or endaural incision to expose the herniation, which is reduced anteriorly, and reconstruction of the external canal using either an autologous or synthetic graft [32,34]. Common autografts used are tragal cartilage, temporalis muscle, and iliac bone crest, while synthetic grafts include collagen mesh, polypropylene mesh, and titanium mesh [4,24,32,35]. Grafts may be secured with sutures or fibrin glue to prevent displacement with TMJ movement post-operatively [18]. On the other hand, the transcanal endoscopic approach is less invasive and performed with an endoscope to raise the skin flap at the anterior wall of the EAC and repair dehiscence with a graft [31].

Conservative treatment may be opted for for patients who are asymptomatic or have mild symptoms. Patients are advised not to chew on the affected side. Analgesics, muscle relaxants, and anxiolytics like clonazepam may be effective in alleviating symptoms [11].

**Figure 4:**
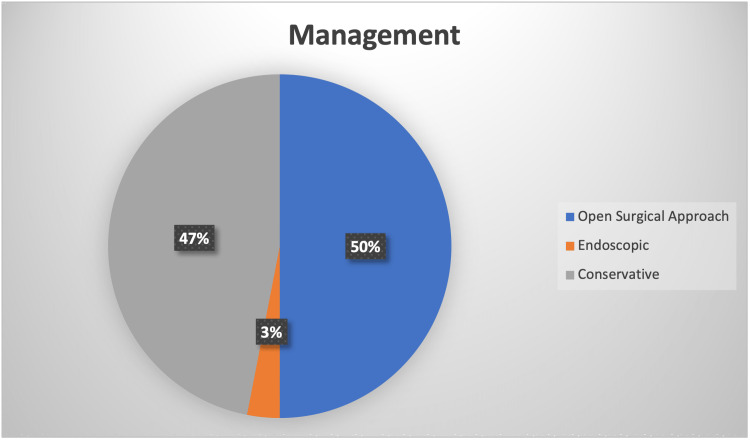
Pie chart showing percentage of choices of management. Image credits: Theresa Teoh.

From this literature review, almost half of the patients (18/37) underwent open surgery. Most of them had an open approach to the preauricular approach, followed by parotidectomy and an extended endaural approach. Only one patient (1/37) underwent endoscopic surgery. The remaining half opted for conservative treatment, as depicted in Figure 4.

The outcome of the surgical intervention is excellent, with all the patients reporting a resolution of symptoms. On the other hand, conservative management (17/37) had mixed outcomes, with only 11.7% (2/17) of patients having complete resolution of symptoms, 29.4% (5/17) had partial improvement of symptoms, and 17.6% (3/17) had persistent symptoms. The remainder of the patients had no records of the outcome.

## Conclusions

The complications of surgery were not mentioned in most of the literature. In addition to these, there are reports of an incidence of surgical site infection and altered sensation during mastication following surgical interventions. Other possible complications that may be encountered include facial nerve paralysis, injuries to the TMJ, tympanic membrane, and middle ear structures, as well as limitations in mouth opening. Despite the overwhelming success of surgical interventions in treating symptomatic persistent foramen of Hushke, it is still early to establish a guideline to manage these patients, as this condition is rare and presents with variable symptoms. In view of limited expertise in surgical management in certain centres, more patients are treated conservatively than surgically. More high-quality studies and a long-term follow-up of the patients may be essential to observe and compare the outcome and recurrence rate of TMJ herniation.
